# Can Environmentally Sustainable Development and Green Innovation of Hotels Trigger the Formation of a Positive Brand and Price Premium?

**DOI:** 10.3390/ijerph18063275

**Published:** 2021-03-22

**Authors:** Jongsik Yu, Junghyun Park, Kyeongheum Lee, Heesup Han

**Affiliations:** 1 Division of Tourism and Hotel Management, Cheongju University, Cheongju-si 28503, Korea; andyjs.yu@gmail.com; 2 College of Hospitality and Tourism Management, Sejong University, Seoul 143747, Korea; junghyun.peterpark@gmail.com (J.P.); heum112311@gmail.com (K.L.)

**Keywords:** environmentally sustainable development (ESD), green innovation, brand image, brand love, brand respect, price premium

## Abstract

This study develops a theoretical framework to describe brand images and customer behaviors in relation to the eco-friendly activities of hotels. These eco-friendly activities were divided into environmentally sustainable development and green innovation. In this study, a survey was conducted on customers who had used a hotel in the past year, and a total of 329 valid samples were obtained. The empirical analysis was conducted using SPSS 22.0 and AMOS 22.0. The empirical analysis results showed that hotels’ eco-friendly activities formed a positive brand image, which in turn had a positive effect on brand love and respect. It was also found that environmental concern, as perceived by customers, did not play a significant moderating role. Therefore, out of a total of eight hypotheses presented in this study, six hypotheses were supported, and two hypotheses were rejected. The findings of this study confirm that hotels’ eco-friendly activities have a positive influence on their performance and provide meaningful insights, based on which strategies for the long-term development and growth of hotels can be established.

## 1. Introduction

Consumers who are directly experiencing global climate change and environmental pollution are more sensitive to environmental issues than ever before. Consumers monitor companies’ business activities and demand corporate responsibility toward the environment throughout the entire business process, including the production, distribution, and consumption of products and services. Additionally, consumers’ awareness of and interest in the environment are becoming more widespread and important, such that they desire to purchase products and services from companies that practice environmentally sustainable and responsible management and development activities [1,2]. That is, a company’s environmental responsibilities and environmentally sustainable management and development practices facilitate the improvement of customer behaviors toward the company [1,3]. These environmental responsibilities and eco-friendly activities serve as predictors for strengthening customer sentiment by forming a positive company image and are important elements in maximizing a company’s performance.

The hotel industry has been growing rapidly over the last few decades, offering a variety of conveniences to customers (e.g., accommodations, meals, banquets, performances, exhibitions, etc.). The growth of the hotel industry provides numerous benefits while also creating several problems. In particular, the various wastes generated due to the nature of the hotel industry, such as greenhouse gas emissions, food waste, water pollution, and household waste are considered to be a major cause of environmental pollution [4]. The adverse impact of the hotel industry on the environment is likely to result in negative consequences for hotels in several aspects, as sustainability and eco-friendly activities are highly valued in the modern tourism and hospitality industry, and consumer demand for eco-friendly products and services is becoming stronger [5,6]. Many studies have found that companies’ pro-environmental policies, attitudes, and eco-friendly products and services have a significant impact on creating positive consumer behaviors [5,7,8]. Therefore, as consumers’ environmental awareness and interest continue to grow, eco-friendly activities of hotels become increasingly relevant and important.

Despite the widespread importance of companies’ eco-friendly activities, little effort has been made to identify the complex connections between hotels’ eco-friendly activities, branding, and price premiums. Specifically, if we examine the previous studies on the eco-friendly activities of hotels, most studies have analyzed the direct impact of the hotel’s eco-friendly activities on the attitude toward the hotel, the image of the hotel, and intention to visit the hotel again [4,9]. However, very few prior studies explained the process of shaping the hotel’s performance through eco-friendly activities, which suggests that the development of a clearer and more specific theoretical framework that can explain the influence of the hotel’s eco-friendly activities on its performance is needed. Therefore, this study was designed to reveal the complex connections between hotels’ eco-friendly activities and the above-mentioned constructs. The purpose of this study is to describe the complex process of explaining a company’s performance through a theoretical model, in which a hotel’s eco-friendly activities create a positive brand image and elicit higher prices. Specifically, this study investigates (1) the impact of eco-friendly activities of hotels, namely, environmentally sustainable development (ESD) and green innovation, on brand image, (2) the impact of brand image on brand love and respect, and (3) whether brand love and respect influence a price premium. Finally, considering that consumers’ environmental concerns vary depending on their individual characteristics, the moderating role of environmental concern in the relationship between hotels’ eco-friendly activities and brand image was investigated.

## 2. Literature Review

### 2.1. ESD and Green Innovation

Excessive development and technological advancement can have adverse effects on human life, including severe environmental damage and worsening environmental pollution. Specifically, global warming, fine dust, natural disasters, climate change, and water pollution can cause human casualties, property damage, and social problems [10]. Consequently, interest in environmental and social concerns continues to grow [11,12]. This phenomenon creates social needs and pressure for ESD, such as reducing environmental pollution, environmentally responsible management, and pro-environmental policies. Therefore, the ESD of a company can be a tool to enhance the company’s brand’s image, love, and respect, and is a necessity for the success of the company.

The World Commission on Environment and Development (WCED) defines environmentally sustainable development as development that satisfies current needs without compromising the rights of future generations [13]. In other words, the main goal of ESD is to develop a sustainable environment through the conservation and protection of nature [14]. ESD can be achieved through environmentally responsible management, such as the conservation of natural and non-renewable resources, responsible waste disposal, preservation of clean air and water, and reduction of hazardous gas emissions [15]. In today’s society, environmental importance continues to be emphasized, thus, recognizing environmental issues and providing environmentally friendly products and services can be the most important strategy for green innovation [16].

Accordingly, many researchers have investigated how companies can develop and implement environmentally friendly strategies to achieve better performance and competitive advantage through green innovation, including the production of eco-friendly products and services [14,17,18]. Many prior studies on the environment commonly argue that companies must establish and implement green innovation strategies, such as pollution prevention, transition to eco-friendly products and services, and development of eco-friendly technologies to preserve and protect the environment. In other words, ESD can be achieved macroscopically through green innovation, which will help companies to develop a positive brand image and promote their growth.

### 2.2. Brand Image

Brand image has been a key concept in consumer behavior research since the 1950s and is still an important element in the marketing field [19]. Brand image is the perception of a brand, reflected by the association of the brand as stored in a consumer’s memory [20]. Brand image can also be considered as a subjective memory that a customer has for a particular subject [21]. Consumers develop either a positive or negative image of a brand through a direct or indirect experience with a product or service. When customers form positive images of a company’s products/services, they are more likely to choose its products/services over those of its competitors [22,23]. Additionally, brand image can positively or negatively affect customers’ intentions and behaviors [24,25]. Because of its importance, many studies have suggested various strategies to improve brand image. In particular, at the present time, when consumers’ awareness of and interest in the environment are increasing, the company’s eco-friendly activities can enhance the corporate image and intention to visit [9,26,27]. Therefore, companies need to attempt to create a positive brand image and recognize that this can improve their performance.

### 2.3. Brand Love and Respect

It is necessary to appeal to consumers’ positive emotions to encourage various consumption-related behaviors because the formation of positive emotions can effectively influence consumer decisions, actions, and loyalty to the brand [28]. Individual decision-making and behaviors are based on logical and complex cognitive processes. However, when making decisions about a brand’s product or service, the contribution of emotional variables can often outweigh that of cognitive variables [29]. Therefore, the significance of emotions is further emphasized because they play a crucial role in the relationship between brands and consumers [30,31].

In an increasingly competitive environment, it is necessary to appeal to consumers’ emotional elements, such as brand love and respect. Brand love refers to emotional attachment to a particular brand and can be described as an emotional link between the brand and customers [32,33]. Brand respect can be considered a positive consumer perception of a particular brand [34]. Brand love and brand respect can maximize the power of relationships between companies and customers and form strong emotional bonds [35]. In particular, the stronger the consumer’s emotional response to social problems such as environmental pollution, the greater significance the consumer will accord to the company’s eco-friendly activities. In other words, the company’s eco-friendly activities are highly likely to develop into love or respect for the brand, which can lead to a variety of positive corporate results. As important emotional elements of customers, brand love and brand respect create long-term outcomes (e.g., increased awareness and loyalty), as well as short-term outcomes (e.g., temporary increase in sales) [31,36]. In addition, brand love and respect can contribute significantly to a company’s performance by inducing preferential behaviors in customers, such as repurchases, recommendations, and payment of high prices [33,35].

### 2.4. Price Premium

Price premium refers to the excess price paid above a fair price [37]. If customers prefer a brand, they may purchase its product or service even if the price is higher than that of another brand [38]. That is, when the value of the product or service as perceived by customers is higher than expected, they accept and pay a price premium. Customers also pay a higher price when they believe that the quality of their preferred brand’s, or a particular brand’s, product or service is relatively higher. This payment of a price premium by consumers indicates their desire for the preferred brand and the quality of the product or service [37]. The price premium can also be seen as a reward to a reputable brand for providing a high level of product or service to consumers [39]. For customers to forgo a competitor’s lower-priced product and pay higher prices instead, a higher value needs to be perceived and provided. Since environmental interests and concerns are growing, the environmental values delivered by a company can encourage customers to pay higher prices and significantly contribute to improving the company’s performance.

### 2.5. Relationships and Study Variables

As customers’ interests and concerns about the environment increase, they consider how the products and services they purchase affect the environment. This increasing customer interest in the environment can induce the company’s eco-friendly activities. In addition, the company’s eco-friendly activities can be a powerful driver that can shape products and services and the overall image of the company. This can influence a company to increase its pro-environmental activities. In addition, a company’s pro-environmental activities can be pivotal in establishing a positive image for the product, service, and company itself. Han et al. [40] suggested that a company’s environmental responsibility activities could improve the quality of its services and promote emotional attachment and word-of-mouth activities. Line and Hanks [9] argued that consumers’ environmental beliefs formed a positive attitude toward green hotels and increased consumers’ intention to pay and revisit. A study by Wang et al. [4] reported that the hotel customers’ interest and concerns about the environment had a positive effect on the overall attitude toward the hotel and further increased their intention to revisit. As such, a hotel’s pro-environmental activities and policies are predictors for developing a positive image and attitude toward the hotel, and further improve financial performance, including the number of revisits by customers. Accordingly, it can be said that the hotel’s eco-friendly activities and policies form a positive image and attitude of the hotel. Furthermore, the hotel’s eco-friendly activities and policies can become a predictor variable that can improve the hotel’s financial performance, such as return visits to the hotel. Therefore, based on prior studies on pro-environmental activities, the following hypotheses were established:

**Hypothesis** **1.** *ESD has a positive effect on brand image.*

**Hypothesis** **2.** *Green innovation has a positive effect on brand image.*

Prior studies on brand image have argued that a positive brand image increases the likelihood of the product or service being chosen and helps to form positive intentions and behaviors of customers [24,25]. Additionally, brand image can predict sentimental attachment to the brand. That is, a positive brand image can form a strong emotional connection between the consumer and a company’s products and services. Song et al. [31] explained that brand image is a powerful factor that can establish customer satisfaction and trust. In other words, a positively formed brand image can maximize emotional attachment to products and services and is a strong predictor of this attachment. Rodrigues and Rodrigues [41] argued that the mystery, sensitivity, and intimacy of a brand as perceived by customers had a positive effect on fostering brand love. This means that, when considering the emotional aspects of customers, creating a positive image of the products and services can induce stronger emotional attachments, which can be expressed as love and respect for the brand. Furthermore, a study by Cho and Fiore [36] explained that brand love and respect are very important factors that can lead to revenue growth by developing a customer’s company awareness and loyalty. This means that sentimental aspects, such as brand love and respect, can maximize company performance when combined with products and services. According to Ba and Pavlou [39], customers are more willing to pay premium prices as their preference for certain products and services increases. Furthermore, Jeong and Jang [37] argued that customers are more likely to pay premium prices when their emotional attachment to certain products and services, that is, the customer desire, is stronger. Based on these prior studies, it is believed that the financial performance of a company can be improved when positive sentimental relationships, such as brand love and respect, are formed between customers and a brand. Therefore, based on these prior studies, the following hypotheses were established:

**Hypothesis** **3.** *Brand image has a positive effect on brand love.*

**Hypothesis** **4.** *Brand image has a positive effect on brand respect.*

**Hypothesis** **5.** *Brand love has a positive effect on price premium.*

**Hypothesis** **6.** *Brand respect has a positive effect on price premium.*

### 2.6. The Modeating Role of Environmental Concern

Environmental concern refers to the extent to which consumers are aware of environmental problems and demonstrate their desire or tendency to participate in solving them [42]. It can also be defined as the extent to which they recognize environmental problems and are willing to contribute personally to solving those problems [43]. In other words, environmental concern is consumers’ emotional response to environmental problems and an important predictor of consumers’ behaviors [44,45]. Such environmental concerns, which affect consumers’ emotional responses and behaviors, vary with individual consumers and may lead to different levels of significance perceived regarding pro-environmental activities of companies. Prior studies have reported that many consumers minimize the consumption of products and services that harm the natural environment, and some further argued that consumers do not tend to consume environmentally irresponsible products or services [5,46,47]. However, Luu’s study [48] found that the impact of pro-environmental activities, such as environmental responsibility activities of a company, on the attitudes and behaviors of organizational members varied with regards to attachment style. That is, the difference in consumers’ emotional responses to pro-environmental activities can represent different consumer responses and behaviors, such as the image perceived, attitude, and behavior toward a company. These results indicate that positive attitudes and behaviors toward a company that practices pro-environmental activities can be formed differently depending on the importance of pro-environmental awareness and behaviors for each consumer. Therefore, based on prior studies on environmental concerns, the following hypotheses were established:

**Hypothesis** **7.** *Environmental concern plays a moderating role in the relationship between ESD and brand image.*

**Hypothesis** **8.** *Environmental concern plays a moderating role in the relationship between green innovation and brand image.*

### 2.7. Research Model

**Figure 1:**
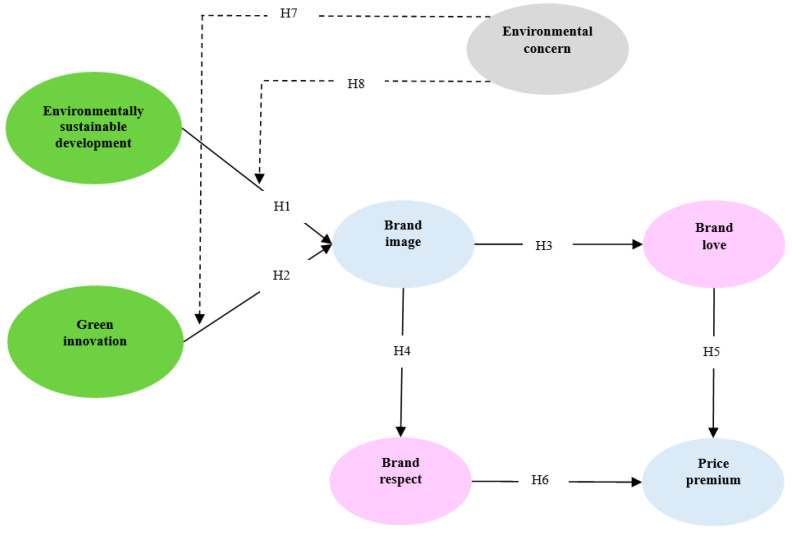
The proposed conceptual framework.

The theoretical framework of this study is designed to examine the impact of the ESD and green innovation of hotels, as perceived by hostel customers, on brand image, brand love, brand respect, and price premium. The verification of the eight hypotheses proposed in the previous section will provide a more specific and clear understanding of the relationship between the variables presented in this study. Specifically, frequency analysis was conducted using SPSS 22.0 (IBM, New York, NY, US) to determine the demographic characteristics of the sample. In addition, confirmatory factor analysis was conducted using AMOS 22.0 (IBM, New York, NY, US) to verify the reliability and validity of the scale, and structural equation modeling was conducted to test the hypotheses. Figure 1 presents the hypotheses and the research model of the study.

## 3. Methods

### 3.1. Measurement and Questionnaire Development

The survey in this study consists of a description of the study, questions on the variables, and questions on demographic characteristics. The feasibility and reliability of the items selected to measure the considered variables were demonstrated in existing studies, which were modified to suit the purpose of this study. A common multi-item questionnaire was used to evaluate the composition of the study, and all measurements were evaluated on a 7-point Likert scale ranging from 1 (highly disagree) to 7 (highly agree). Specifically, four questions on ESD based on Bansal [49], and four questions on green innovation based on Song and Yu [18] were used. In addition, three questions on brand image based on Baloglu and McClearly [50], as well as three questions on brand image and respect based on Carroll and Ahuvia [51] and Han et al. [52] were included. Finally, three questions on price premiums based on Miller and Mills [53] were used. For the purposes of this study, a pretest was conducted on researchers and operators in the hotel and tourism fields to modify and supplement the initial questionnaire. The pretest was completed by a total of seven people, including two university professors, two graduate students, and three staff members, and, thus, the comprehensiveness of the survey was improved. The pretest was conducted on a total of seven persons (e.g., two university professors, two graduate students, and three hotel officials), and the appropriateness of the questionnaire’s content, clarity of sentences, and consistency with the purpose of the study were verified through the pretest. As a result, the completeness of the survey content could be improved.

### 3.2. Data Collection and Sample Characteristics

The data used in this study’s empirical analysis were collected through an Internet research agency that uses web-based systems located in South Korea. The data used for the empirical analysis in this study were derived via a convenience sampling method. The purpose of this study was explained to the survey participants along with a guarantee that the data collected would never be used for any other purpose. It was also stated that the personal information of the survey participants would be kept confidential. Therefore, the questionnaire for this study was designed such that all respondents agreed with the purpose of this study and participated voluntarily. In addition, the survey was conducted with customers who had used a hotel within the past year. A total of 337 responses were obtained through this method, from which eight responses that were not answered accurately or completely were excluded. Therefore, the empirical analysis was conducted using a total of 329 responses. First, a frequency analysis was conducted to identify the demographic characteristics of the survey participants. The analysis indicated that 52.9% of the participants were men and 47.1% were women. Regarding age distribution, 33.1% were in their 20s, 44.7% in their 30s, 18.5% in their 40s, and 3.7% in their 50s or older. Regarding level of education, 2.7% had graduated from high school, 23.1% from college, 58.7% from university, and 15.5 % from graduate school or higher. Lastly, regarding income distribution, 14.3% earned an annual income of $40,000 or less, 55.6% earned between $40,000 and $60,000, 25.2% earned between $60,000 and $80,000, and 4.9% earned $80,000 or more. Table 1 presents the analysis results.

## 4. Results

### 4.1. Measurement Model Results

Anderson and Gerbing [54] suggested that confirmatory factor analysis is the most useful method of analysis to verify the reliability and validity of a scale. Therefore, to verify the reliability and validity of the scale used in this study, a confirmatory factor analysis was performed using the maximum likelihood estimation method. The analysis showed that the model fit of the measurement model used in this study was χ^2^ = 361.967, df = 209, *p* < 0.001, χ^2^/df = 1.732, RMSEA = 0.047, CFI = 0.974, and TLI = 0.968, which was statistically acceptable. Next, the reliability of the measured items was evaluated using standardized regression weights. All items ranged from 0.661 to 0.926, which was above the standardized regression weight of 0.5. Therefore, all measurement items presented in this study were confirmed to be reliable. Subsequently, the average variance extracted (AVE) and composite reliability (CR) were verified to confirm convergent validity and internal consistency. If the AVE value is 0.5 or higher, and the CR value is 0.7 or higher, the internal consistency and convergent validity of a measurement variable can be considered appropriate [55]. The AVE values of the measurement variables ranged from 0.624 to 0.786, and the CR values ranged from 0.864 to 0.944, which confirmed the convergent validity and internal consistency of the measurement variables, respectively. Finally, discriminant validity analysis was performed to confirm the distinction between the constructs. Discriminant validity can be assumed appropriate if the AVE value is greater than the squared value of the correlation coefficient [55]. The analysis results indicated that the AVE value was greater than the squared value of the correlation coefficient, which confirmed the discriminant validity between the variables. Table 2 presents the analysis results.

### 4.2. Structural Model Results and Hyporheses Testing

In this study, the conceptual characteristics and proposed hypotheses were validated through structural equations using the maximum likelihood method. The model fit of this study was found to be appropriate, with χ^2^ = 464.140, df = 143, *p* < 0.001, χ^2^/df = 2.847, RMSEA = 0.075, CFI = 0.942, and TLI = 0.932. Following this, the first six hypotheses proposed in this study were validated, and the results are as follows: ESD was found to have a significant effect on brand image (β = 0.638, *p* < 0.01), and green innovation also had a significant effect on brand image (β = 0.141, *p* < 0.05). Thus, Hypotheses 1 and 2 were supported. Next, the effects of brand image on brand love and brand respect were validated. The results showed that brand image had a significant effect on both brand love (β = 0.787, *p* < 0.01) and brand respect (β = 0.763, *p* < 0.01). Therefore, Hypotheses 3 and 4 were supported. Finally, the effects of brand love and brand respect on price premiums were validated. Brand love was found to have a significant effect on price premium (β = 0.532, *p* < 0.01), and brand respect also had a significant effect on price premium (β = 0.489, *p* < 0.05). Thus, Hypotheses 5 and 6 were supported.

The use of a mediating framework can be of great help in understanding the complex relationships between study components within a theoretical model [56]. Therefore, the indirect effects were validated using bootstrapping to help understand the complex relationships in the model. According to the analysis results, ESD had a significant indirect effect on price premium (β_environmentally sustainable development–brand image–brand love & respect–willingness to pay =_ 0.505, *p* < 0.01), brand love (β_environmentally sustainable development–brand image–brand love =_ 0.501, *p* < 0.01), and brand respect (β_environmentally sustainable development–brand image–brand respect =_ 0.487, *p* < 0.01). However, green innovation indicated no significant indirect effect on price premium (β_green innovation–brand image–brand love & respect–willingness to pay =_ 0.112, *p* > 0.05), brand love (β_green innovation–brand image–brand love =_ 0.111, *p* > 0.05), and brand respect (β_green innovation–brand image–brand respect =_ 0.108, *p* > 0.05). Therefore, the mediating role of brand image, brand love, and brand respect was partially demonstrated within the theoretical framework presented in this study. Table 3 present the results of these analyses.

### 4.3. Structural Invariance Model Assessment

In this study, an invariance test was performed to verify the mediating role of environmental concern in relation to the effects of ESD and green innovation on brand image. Hypotheses 7 and 8 were validated by dividing the data used in the empirical analysis into a high environmental concern group (*n* = 195) and a low environmental concern group (*n* = 134). First, the mediating role of environmental concern in relation to the effect of ESD on brand image was validated. As a result, environmental concern did not appear to have a mediating role that was statistically significant (Δχ^2^(1) = 3.145, *p* > 0.05). Next, the mediating role of environmental concern in relation to the effect of green innovation on brand image was validated, which also indicated that environmental concern had no statistically significant mediating role (Δχ^2^(1) = 1.154, *p* > 0.05). Therefore, Hypotheses 7 and 8 were rejected. These are meaningful results as they imply that ESD and green innovation are very important in each of the presented relationships, regardless of environmental concerns. Table 4 present the results of these analyses.

## 5. Discussion and Implications

The most important finding in this study is that ESD and green innovation of hotels, as perceived by consumers, can improve brand image, as well as brand love and respect. Moreover, ESD and green innovation help to create a price premium that can maximize a hotel’s financial performance. In other words, ESD and green innovation are variables that can develop brand assets, such as the hotel’s brand image, love, and respect, which serve as important means of improving the hotel’s financial performance. Such improvements in positive brand assets and financial performance can be a major driver for the future growth of hotels. The results of this study help us understand the formation of a positive brand based on ESD and green innovation of hotels and provide measures to maximize hotels’ financial performance, especially from a long-term perspective.

These results confirm the significant role of a hotel’s pro-environmental activities, as recognized by consumers, in relation to the importance of the constructs presented in this study. Many prior studies have also reported that companies’ environmentally friendly activities, such as environmental practices and environmental corporate social responsibility, have a positive impact on their performance [14,17,18]. In other words, pro-environmental activities, such as ESD and green innovation of hotels, are very important factors that can maximize business performance. Next, brand image was found to have a very positive impact on brand love and respect, which in turn could motivate consumers to pay a large amount of money at hotels. These results are consistent with those of existing studies that highlight the importance of emotional factors in eliciting positive customer decisions for a particular brand or company [29,57]. In other words, in the hotel industry, the development of consumers’ brand love and respect is the key element in improving the performance of hotels based on the emotional aspect of the decision-making process of hotel customers. As the study results indicate, it is necessary to strengthen the positive emotional connection between consumers and hotel brands to improve hotel performance.

In this study, the environmental concern, as perceived by consumers, did not show any statistically significant difference between the high and low environmental concern groups regarding the relationship between the pro-environmental activities of hotels, such as ESD and green innovation, and brand image. Therefore, Hypotheses 7 and 8 were rejected. These results provided meaningful insights. First, regardless of the extent of environmental concern held by consumers, consumers think positively about the pro-environmental activities practiced by hotels. Second, despite the finding that pro-environmental activities positively affect hotel performance regardless of the extent of environmental concern, the high environmental concern group appears to form a more positive brand image of hotels that are more active in pro-environmental activities. This suggests that consumers with high environmental concern tend to show relatively positive behaviors toward hotels that practice pro-environmental activities. This is demonstrated by the values of the high environmental concern group (environmentally sustainable development β = 0.645/green innovation β = 0.123), which are higher than that of the low environmental concern group (environmentally sustainable development β = 0.509/green innovation β = 0.522).

This study makes a meaningful contribution to extant literature since the strategy to maximize hotel performance mentioned in prior studies has been extended to the field of pro-environmental activities. In particular, this study revealed that the environmentally friendly activities of hotels (e.g., eco-friendly product use, waste disposal systems, water conservation, and carbon gas reduction policies) could establish a positive image for hotels and further strengthen the emotional aspect of the decision-making process of customers. It was also demonstrated that a price premium for hotel products and services could be created to maximize financial performance. Therefore, the results of this study support prior studies on brand image of hotels and financial performance, while successfully addressing the insufficiencies of those studies. These findings can provide hotel operators with future directions for improving their performance. Specifically, hotel operators need to be more committed to implementing pro-environmental activities (e.g., recycling policies, waste management, energy conservation, and restrictions on the use of disposable products). They should stop replacing blankets in the rooms on a daily basis, regardless of customers’ intentions, but instead replace room supplies as requested by customers. In addition, the use of disposable products should be discouraged, while bath products and cleaning supplies that minimize water pollution should be promoted. Such efforts by hotel operators to reduce environmental pollution can form a positive image for hotels and maximize their financial performance.

Despite the meaningful findings of this study, there were several limitations. First, since the pro-environmental activities covered in this study are only those of the hotel industry, the generalization of the findings is limited. Second, since the survey targeted only Korean nationals, the application for other countries, races, or continents is limited. Third, since the participants in the study were limited to hotel customers who used a hotel in the last year, the study does not reflect the opinions of potential users who have not used a hotel in the recent past. Therefore, the study will be more meaningful if the target sample is expanded beyond the hotel industry, and the study subjects include other regions, cultures, and potential users with no previous experience of using a hotel.

## 6. Conclusions

Various environmental problems continue to occur across the world, and not only individuals but also many companies are conscious of and interested in environmental problems. Consumers who are interested in environmental issues want to actively support companies’ eco-friendly activities and purchase the products and services of companies that are active in eco-friendly activities. Therefore, in this study, we examined the effects of the hotel’s eco-friendly activities on the overall brand image, brand love and respect, and price premium of the hotel in a situation in which awareness and interest in environmental issues continue to increase. Specifically, the hotel’s eco-friendly activities were presented separately as environmentally sustainable development and green innovation. It was found through the empirical analysis that the sustainable development and green innovation of the hotel had a positive effect on the brand image, brand love and respect, and price premium. In addition, it was confirmed that the brand image and the brand love and respect played partial mediating roles within the theoretical framework proposed in this study. In addition, the group with high environmental concern was found to accord more significance to the hotel’s eco-friendly activities. Therefore, it can be said that the study was successful in achieving its purpose and provided very meaningful implications.

## Figures and Tables

**Table 1:** The respondents’ demographic information (*n* = 329).

Characteristics		Frequency	%
Gender	Male	174	52.9%
	Female	155	47.1%
Age	20–29	109	33.1%
	30–39	147	44.7%
	40–49	61	18.5%
	Over 50	12	3.6%
Education	High school graduate	9	2.7%
	2-year college	76	23.1%
	College graduate	193	58.7%
	Graduate degree	51	15.5%
Annual household income	$40,000 or less	47	14.3%
	$40,001–$59,999	183	55.6%
	$60,000–$79,999	83	25.2%
	$80,000 or above	16	4.9%

**Table 2:** Measurement model assessment and correlation (*n* = 329).

Constructs	(1)	(2)	(3)	(4)	(5)	(6)	(7)
(1) Environmentally sustainable development	1.000						
(2) Green innovation	0.641 ^a^ (0.410) ^b^	1.000					
(3) Brand image	0.554 (0.306)	0.435 (0.189)	1.000				
(4) Brand love	0.631 (0.398)	0.702 (0.492)	0.591 (0.349)	1.000			
(5) Brand respect	0.657 (0.431)	0.532 (0.283)	0.592 (0.350)	0.619 (0.383)	1.000		
(6) Price premium	0.711 (0.505)	0.615 (0.378)	0.653 (0.426)	0.745 (0.555)	0.733 (0.537)	1.000	
(7) Environmental concern	0.634 (0.401)	0.709 (0.502)	0.504 (0.254)	0.735 (0.540)	0.610 (0.372)	0.697 (0.485)	1.000
Mean	3.864	4.054	3.433	3.905	3.661	3.738	3.925
SD	0.748	0.688	1.059	0.731	0.990	0.796	0.678
Composite reliability (CR)	0.868	0.944	0.893	0.882	0.917	0.905	0.864
Average variance extracted (AVE)	0.624	0.760	0.736	0.714	0.786	0.761	0.679

Note. Goodness-of-fit statistics: χ^2^ = 361.967, *df* = 209, *p* < 0.01, χ^2^/*df* = 1.732, RMSEA = 0.047, CFI = 0.974, TLI = 0.968. ^a^ Correlations. ^b^ Squared correlations.

**Table 3:** The structural model estimation (*n* = 329).

Hypothesized Paths				Coefficients	*t*-Values
H1	Environmentally sustainable development	→	Brand image	0.638	7.026 **
H2	Green innovation	→	Brand image	0.141	1.841 *
H3	Brand image	→	Brand love	0.787	13.383 *
H4	Brand image	→	Brand respect	0.763	14.151 **
H5	Brand love	→	Price premium	0.532	9.597 **
H6	Brand respect	→	Price premium	0.489	9.554 *
Indirect effect: β_environmentally sustainable development→brand image→brand love & brand respect→price premium_ = 0.505 ** β_green innovation→brand image→brand love & brand respect→price premium_ = 0.112 β_brand image→brand love & brand respect→price premium_ = 0.792 ** β_environmentally sustainable development→brand image→brand love_ = 0.501 ** β_environmentally sustainable development→brand image→brand respect_ = 0.487 ** β_green innovation→brand image→brand love_ = 0.111 β_green innovation→brand image→brand respect_ = 0.108				Explained variance: R^2^ (brand image) = 0.560 R^2^ (brand love) = 0.619 R^2^ (brand respect) = 0.583 R^2^ (Price premium) = 0.836	

Note. Goodness-of-fit statistics for the structural model: χ^2^ = 464.140, *df* = 143, *p* < 0.001, χ^2^/*df* = 2.847, RMSEA = 0.075, CFI = 0.942, TLI = 0.932. * *p* < 0.05, ** *p* < 0.01.

**Table 4:** The invariance tests for structural models (*n* = 329).

Paths	High Environmental Concern (*n* = 195)		Low Environmental Concern (*n* = 134)		Baseline Model (Freely Estimated)	Nested Model (Constrained to Be Equal)
	β	*t*-Values	β	*t*-Values		
H7a: ESD → BI	0.645	4.835 **	0.509	4.330 **	χ^2^ (340) = 656.924	χ^2^ (341) = 660.069 ^a^
H7b: GI → BI	0.123	1.202	0.061	0.522	χ^2^ (340) = 656.924	χ^2^ (341) = 658.078 ^b^
Chi-square test: ^a^ Δχ^2^ (1) = 3.145, *p* > 0.05 ^b^ Δχ^2^ (1) = 1.154, *p* > 0.05	Hypotheses testing: H7: Not supported H8: Not supported			Goodness-of-fit statistics for the baseline model: χ^2^ = 656.924, *df* = 340 *p* < 0.01, χ^2^/*df* = 1.932, RMSEA = 0.053, CFI = 0.911, IFI = 0.912, TLI = 0.901 * *p* < 0.05, ** *p* < 0.01		

Note. ESD: environmentally sustainable development, GI: green innovation, BI: brand image.

## Data Availability

The dataset used in this research are available upon request from the corresponding author. The data are not publicly available due to restrictions i.e., privacy or ethical.
